# Ultrasound as a Biomarker in Rheumatic Diseases

**DOI:** 10.3390/diagnostics10110933

**Published:** 2020-11-10

**Authors:** Kai Quin, Hareth M. Madhoun

**Affiliations:** Department of Internal Medicine, Division of Rheumatology and Immunology, Ohio State University College of Medicine, Columbus, OH 43210, USA; hareth.madhoun@osumc.edu

**Keywords:** ultrasound, Doppler, synovitis, rheumatologic disease, rheumatoid arthritis, gout, giant cell arteritis, polymyalgia rheumatica, Sjogren’s syndrome, calcium pyrophosphate deposition disease

## Abstract

Rheumatic diseases are a heterogeneous group of disorders which often affect the musculoskeletal system. Given the lack of definitive testing, there are limited diagnostic tools at clinicians’ disposal. Over the recent decades, ultrasonography has gained widespread use within rheumatology due to its accessibility, safety, and relatively low cost. This review describes the clinical utility of ultrasound as a biomarker in the diagnosis and management of several rheumatic diseases.

## 1. Introduction

Rheumatic diseases are a heterogeneous collection of disorders which affect the musculoskeletal system, causing pain, structural deformities, and loss of function. Diagnosis relies on a combination of clinical history, physical examination, imaging, and serologic markers [1]. There are few definitive diagnostic tools, thus patient specific considerations and the clinical acumen of the practitioner cannot be overstated. In recent decades, musculoskeletal ultrasound (MSUS) has become increasingly prevalent in the diagnosis and management of rheumatic diseases [2]. Point-of-care MSUS reduces utilization of healthcare resources compared to magnetic resonance imaging (MRI) through cheaper cost, shorter time to diagnosis [3], and fewer clinic visits [4]. Furthermore, ultrasound (US) does not emit ionizing radiation, nor are there any absolute contraindications, given that it does not involve a magnetic field or gadolinium contrast. Therefore, it can be employed in patients with metallic implants and chronic kidney disease. This review will provide an overview of US physics, rheumatic diseases, and common MSUS imaging findings.

## 2. Background

Sound is transmitted as mechanical energy in the form of vibration, whereas light is an electromagnetic energy. Therefore, sound requires a medium through which to propagate, while light can travel through a vacuum. Different substances allow different transmission speeds and have different acoustic impedance, which is a measure of the opposition that a structure presents to vibrational energy. When sound waves reach an interface between two materials possessing different acoustic impedance, a portion of the energy will be reflected while the rest propagates. The greater the difference in the impedance, the more energy will be reflected [5]. In 1794, Italian physicist Lazzaro Spallanzani discovered that bats use US to navigate via echolocation as opposed to light and vision. This introduced the concept that sound, like light, can be used to identify objects and their characteristics. Physicist Lord Rayleigh’s 1877 textbook “Theory of Sound” subsequently laid the mathematical foundation for acoustics [6].

Audible frequencies range from 30 to 20,000 Hertz. US is defined as high frequency sound waves above the range of human hearing, and the frequencies commonly used in diagnostic US are between 5 and 16 megahertz (MHz). Higher frequencies offer higher resolution, but lower depth of penetration. On the other hand, lower frequencies penetrate deeper into a medium, but possess lower spatial resolution. For MSUS, the frequencies used must be able to differentiate small anatomic structures, such as tendons and ligaments of the hand. They must also penetrate deep to assess bony articulations such as the hip. For small joint and superficial soft tissue visualization, a linear broadband transducer with frequencies of 5 to 13 MHz is often used. A curvilinear transducer with frequencies of 2 to 6 MHz is appropriate for large and deep joints [7].

The piezoelectric effect was discovered in 1880 by Pierre and Jacques Curie, who determined that an electric current applied across a crystal produced vibrations that would generate sound waves [8]. Piezoelectric transducers then allowed for applications in medicine with the development of A-mode, which is the simplest type of US that provides a linear scan through the body. B-mode, otherwise known as 2D mode, uses a series of piezoelectric crystals in a linear array. This scans a plane through the body producing a two-dimensional image [9]. Medical use of US began as a therapeutic tool for rheumatism and Parkinson’s disease, then later as a cornerstone of diagnostic imaging. Modern use of US is widespread across different specialties, including obstetrics and gynecology, rheumatology, endocrinology, cardiology, pulmonology, emergency medicine, and critical care.

Doppler sonography is a powerful tool that can be used to visualize movement. In medical applications, this is normally used to measure blood flow. Color Doppler provides information about the direction and speed of blood flow, but has a relatively limited detection threshold. Power Doppler sonography is a newer technique that uses signal amplitude to identify motion, rather than velocity and direction. This allows for greater detection of smaller blood vessels with lower flow velocities.

MSUS provides dynamic real-time assessment of articular, peri-articular, and soft tissue structures. Improvements in spatial resolution and tissue contrast have led to increased sensitivity and specificity, particularly in the diagnosis of inflammatory arthritides [10]. Newer developments have further expanded its use to vascular and exocrine tissues. This frequently offers clinicians crucial clinical information at the point of care that is otherwise unattainable by history and physical examination [11].

## 3. Musculoskeletal Ultrasound Artifacts

US images are created by the analysis and interpretation of sound waves that are reflected by a difference in the impedance between two adjacent media. The greater the difference, the higher the percentage of reflected vibrational energy, which in turn creates a hyperechoic or brighter signal. The interface between soft tissue and bone is an example of extreme difference in impedance. It produces a very hyperechoic signal at the bony surface with an anechoic area underneath it. This artifact is called acoustic shadowing, and also allows for the identification of calcifications within soft tissues. An opposite effect called posterior acoustic enhancement occurs when US waves pass through low-density media such as joint effusions or cysts, and produce higher echogenicity beneath the structure compared to surrounding tissues. Posterior reverberation occurs at a smooth and flat surface such as the cortex of a long bone or a metallic implant. The sound wave reflects back and forth between the object and the transducer, creating hyperechoic linear signals deep into the image. Ring-down artifact describes a variant of this phenomenon in which there is a continuous signal beneath the surface.

Anisotropy is an important artifact when evaluating tendons, ligaments, and to a lesser extent muscle. When these tissues are positioned perpendicular to the US beam, it produces a clear image. For tendons and ligaments, the internal fibrils can often be seen. However, if the US beam is positioned at an angle to the long axis, the tissue becomes hypoechoic with loss of normal fibrillar appearance. Even a small change in the viewing angle can cause loss of echogenicity [12]. Sonographers can correct for anisotropy in the short axis by toggling the transducer along the long axis of the structure. When viewing in the long axis, a “heel-toe” maneuver can be employed. Awareness of this effect is important in order to minimize misdiagnosis of tendon pathologies. However, anisotropy can sometimes be useful in identifying tissues such as the median nerve, which appears similar but displays less anisotropy compared to its neighboring wrist flexor tendons.

## 4. Rheumatoid Arthritis

Rheumatoid arthritis (RA) is a chronic systemic autoimmune disease affecting 1.28–1.36 million people in the United States [13]. The pathophysiology of RA is poorly understood, but is believed to be the result of inherited susceptibility and environmental triggers that lead to overactivity and dysregulation of the adaptive and innate immune system [14]. Its most common clinical manifestation is inflammatory arthropathy, which typically presents in a symmetric pattern of polyarthritis or oligoarthritis. RA most commonly involves small and medium joints, though large joints may occasionally be affected. Patients often report morning stiffness greater than one hour, and improvement of pain with increased physical activity [15]. On physical examination, inflammatory arthritis is characterized by joint swelling, effusion, tenderness to palpation, warmth, or erythema. In the later stages of the disease, joint deformities can be evident. Swan-neck deformities affect the fingers, and cause proximal interphalangeal joint (PIP) hyperextension and distal interphalangeal (DIP) joint flexion [16]. Boutonniere deformities, on the other hand, lead to PIP flexion and DIP hyperextension [17]. Subluxation of the metacarpal phalangeal joints cause ulnar deviation.

Like many rheumatologic diseases, there is no definitive diagnostic test for RA. Clinical history, serological abnormalities, physical examination, and imaging can help establish a diagnosis. The 2010 American College of Rheumatology/European League Against Rheumatism collaborative initiative devised a set of score-based diagnostic criteria, in which the number and distribution of affected joints, presence of rheumatoid factor or anti-citrullinated peptide antibody, elevation of C-reactive protein or erythrocyte sedimentation rate, and duration of symptoms greater than six weeks are each allocated a numerical score. If no alternative diagnosis can be identified, a diagnosis of RA can be made if the total score is six or greater out of a possible ten [18].

**Figure 1:**
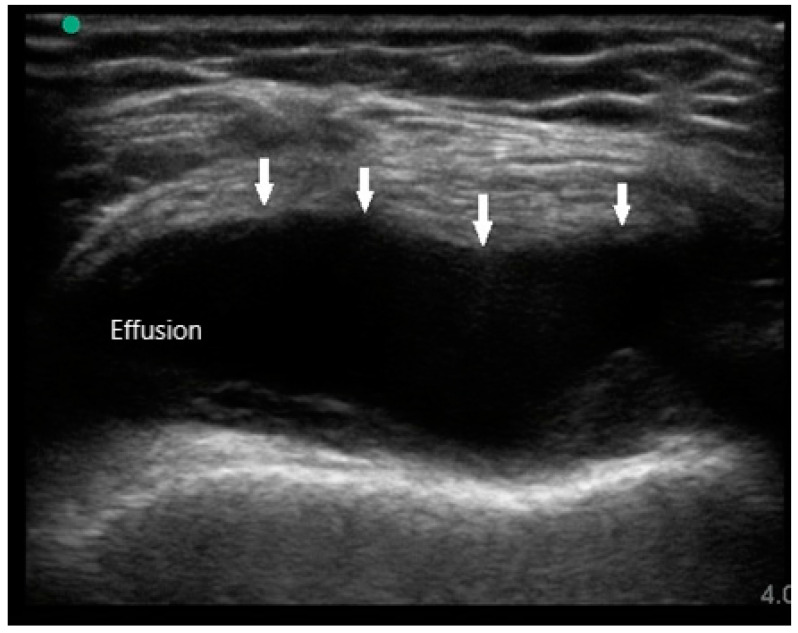
Knee effusion (arrows) in suprapatellar short axis view.

MSUS is primarily used to assist in diagnosis by identifying the presence of synovitis in symptomatic joints, especially if physical examination is difficult or equivocal. The sonographic features of RA are joint effusion, synovial hypertrophy, bone erosions, tenosynovitis, and hyperemia. Joint effusions appear as compressible hypoechoic or anechoic material in the joint space, often detected in the dependent recesses of the synovium. For large joints such as knees, effusions are most easily seen in the suprapatellar fossa in either long or short axis (Figure 1). In small joints such as PIPs, effusions can be detected over the joint on the palmar aspect deep to the flexor tendon. While the presence of joint effusion does not prove the presence of inflammatory arthritis, it does supplement the physical exam and indicate the potential need for arthrocentesis and synovial fluid analysis.

**Figure 2:**
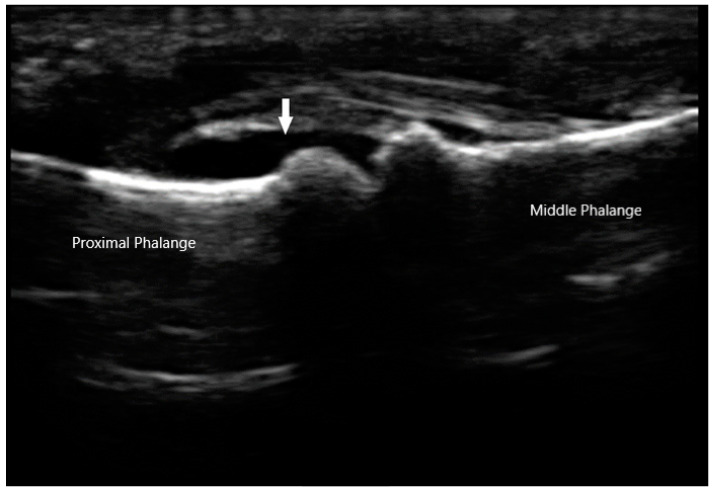
Synovial effusion (arrow) of proximal interphalangeal joint.

**Figure 3:**
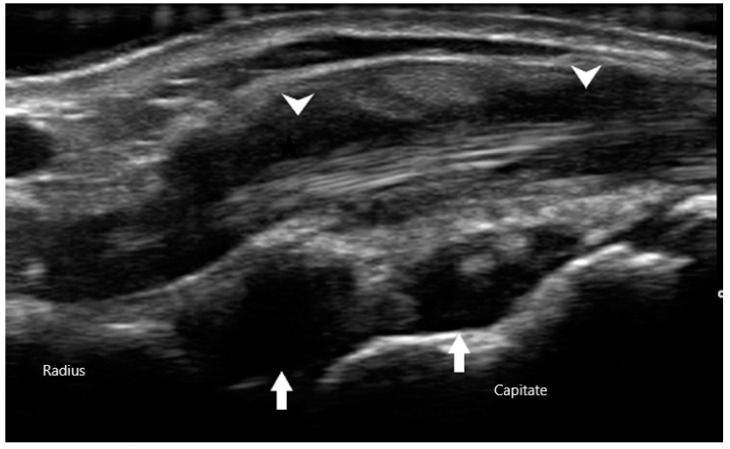
Synovial hypertrophy (arrows) of radiocarpal joint with tenosynovitis (arrowheads) of extensor tendon.

The Outcomes Measures in Rheumatology (OMERACT) group has established a consensus-based system to define and grade synovitis identified by greyscale and power Doppler [19]. Synovial hypertrophy is visualized as abnormal hypoechoic material in intra-articular tissue that is non-displaceable and non-compressible (Figure 2). In order to distinguish it from an effusion, sonographers can apply pressure on the transducer in order to evaluate for compressibility. It is graded on a scale of 0 to 3. Grade 1 is defined as minimal synovial thickening without protrusion over the adjacent bone. Grade 2 is more extensive synovial thickening with bulging over the bone. Grade 3 describes extensive thickening with extension beyond the joint. Angiogenesis is another hallmark of synovitis, and this hypervascularity can often be detected by increased power Doppler signal, which can also be graded from 0 to 3 [20]. Grade 0 refers to lack of Doppler activity. Grade 1 is mild activity. Grade 2 and 3 are defined as Doppler signals covering <50% and >50% of the joint area respectively. Tenosynovitis is characterized by hypoechoic material or fluid within tendon sheath (Figure 3).

**Figure 4:**
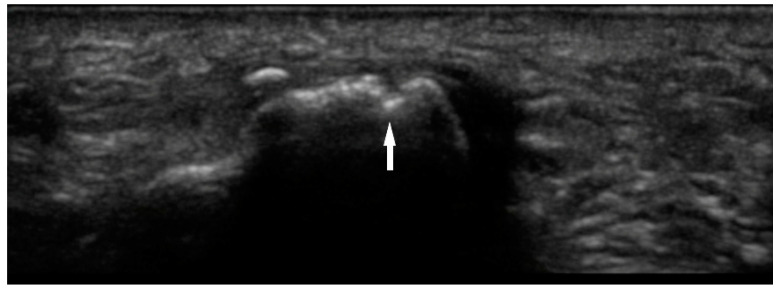
Bone erosion of 1st metatarsal phalangeal joint with cortical discontinuity (arrow).

Bone erosions are the result of chronic synovial inflammation, leading to increased periarticular bone resorption. They can be identified on several different imaging modalities, including radiography, MRI, and US. MRI is the most sensitive method of detecting these lesions, but it is not frequently used due to limited accessibility and high cost. While radiography is ubiquitous, it may not be as sensitive as MSUS. Erosions are defined on US as discontinuity of cortical bone identified in two perpendicular or orthogonal planes (Figure 4) [19]. A 2000 study by Wakefield et al. demonstrated that MSUS was more sensitive in evaluation of erosions than radiography. This discrepancy was especially evident in early disease, where sonography detected 6.5 times more lesions in 7.5 times more patients. In late stage of the disease, the difference was 3.4 times and 2.7 times respectively. [10]

Despite arthritis being the predominant element, extra-articular manifestations such as cardiovascular and pulmonary involvement are the leading cause of mortality and morbidity [21,22]. Therefore, early diagnosis and treatment is crucial in order to prevent disease progression and reduce cardiovascular mortality [23]. US has been shown to aid in the diagnosis of RA, even when active synovial inflammation is not detected clinically [24]. Furthermore, subclinical synovitis detected in patients thought to be in remission is correlated with progression of structural damage [25]. Sonographic detection of synovitis underscores the importance of the use of MSUS during clinical decision making.

## 5. Spondyloarthropathy

Spondyloarthropathies (SpA) are a group of related autoimmune diseases, characterized by its association with human leukocyte antigen B27 (HLA-B27). They include ankylosing spondylitis, psoriatic arthritis, reactive arthritis, inflammatory bowel disease related arthritis, and undifferentiated spondyloarthritis [26]. The estimated prevalence of axial SpA in the United States is 1.0 to 1.4 percent, though there exists significant geographic variation worldwide due to the differences of HLA-B27 prevalence [27].

Similar to RA, inflammatory arthritis is the defining feature of SpA. However, there are several key differences. SpA frequently affects the sacroiliac (SI) and intervertebral joints. With the exception of the cervical spine, the axial skeleton is typically spared in RA. Bone marrow edema on MRI is the earliest imaging evidence of SI joint inflammation, and may progress to erosions [28]. Vertebral and SI joint ankylosis are late stage sequelae of ankylosing spondylitis if the disease is untreated. US can be used to guide therapeutic injections into the SI joints, but is inadequate for complete assessment as only the anterior and posterior margins are visible [29].

**Figure 5:**
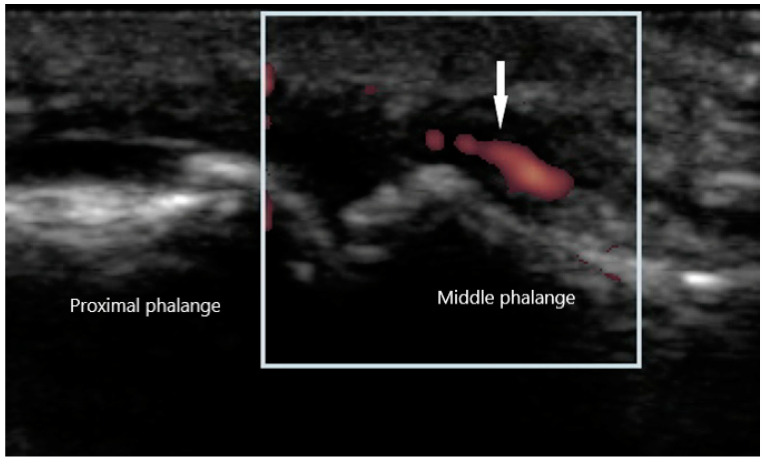
Synovitis of proximal interphalangeal joint with power Doppler signal (arrow).

**Figure 6:**
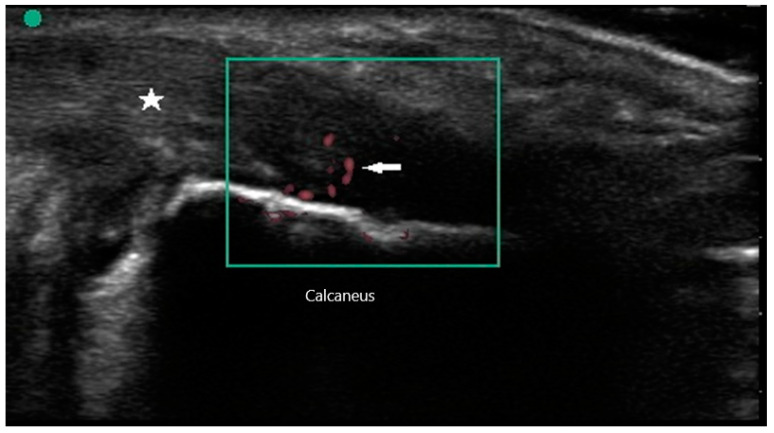
Enthesitis of Achilles tendon (star) with power Doppler signal (arrow).

**Figure 7:**
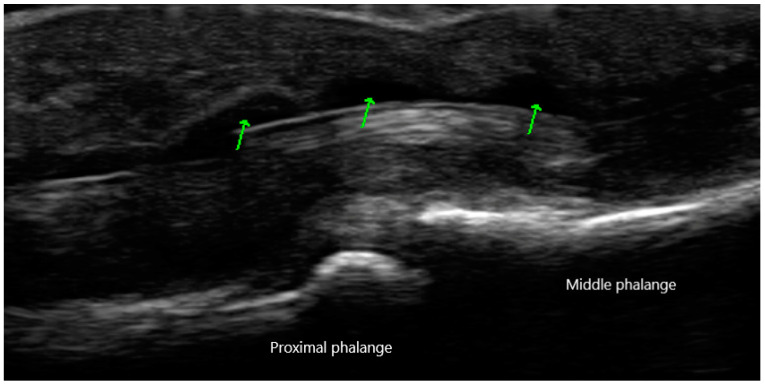
Dactylitis of digit with diffuse tenosynovitis of flexor tendon (arrows) with associated soft tissue swelling.

Peripheral joint involvement can be similar between SpA and RA, though the former more commonly presents as asymmetric oligo-arthritis. SpA, especially psoriatic arthritis, may also affect the DIP joints, which is a feature never seen in RA. Furthermore, tendinous and peritendinous structures are more often affected, as is seen in dactylitis and enthesitis. Sonographic characteristics of peripheral joint inflammation in SpA are also similar to that of RA, both in greyscale and power Doppler (Figure 5). Joint effusions are compressible, displaceable, and hypoechoic substances within the joint space, while synovitis is a non-compressible, non-displaceable thickening of the synovium. Hyperemia on power Doppler may also be present. Enthesitis is inflammation of the insertion of ligament, fascia, or tendon into bone. The most commonly affected entheses are the Achilles tendon (Figure 6), plantar fascia, lateral humeral epicondyle, greater trochanter, and pes anserinus [30]. On physical examination, patients may exhibit pain on palpation or swelling of these structures. Sonographic findings reveal hypoechoic or thickened insertion of the tendon 2 mm or less from the bony cortex. Doppler signal, bone spurs at tendinous insertion known as enthesophytes, calcifications, or erosions may be present [31]. Dactylitis, or “sausage” digits (Figure 7), is a well described yet rare finding in SpA, characterized by diffuse tenosynovitis, synovitis, and enthesitis [32].

## 6. Sjogren’s Syndrome

Clinical utility of US is not limited to articular diseases. Salivary gland ultrasound (SGUS) has made headway into the diagnosis of Sjogren’s syndrome (SS), a slowly progressive autoimmune disease, causing lymphocytic infiltration into lacrimal, parotid, and salivary glands. Sicca symptoms include xerophthalmia, xerostomia, and parotid gland enlargement [33]. Diagnosis relies on objective measures of exocrine gland activity. Tear production is quantified by Schirmer’s test, and saliva production by sialography, sialometry, and scintigraphy. However, the gold standard is minor salivary gland (MSG) biopsy, where >50 lymphocytes around the salivary gland acini with ≥1 focus/4 mm^2^ is 64–86% sensitive and 90–100% specific for SS [34]. Due to the invasive nature of a salivary gland biopsy, it is not consistently pursued by clinicians.

SGUS is an inexpensive, safe, and non-invasive diagnostic test with favorable diagnostic accuracy [35]. An international panel of experts has devised a scoring system based on sonographic features found within the parotid and submandibular glands. Core items include parenchymal echogenicity, heterogeneity, presence of hypoechoic, and hyperechoic areas, hyperechoic bands, abnormal lymphadenopathy, and calcifications [36]. Parenchymal echogenicity was assessed and classified as normal or fibrotic. Homogeneity was classified as normal or abnormal. The presence of hyperechoic bands was graded as none, <50%, or ≥50% of the parenchyma. Hypoechoic areas were counted, and the largest was measured in millimeters. They were also graded as <25%, 25–50%, >50%, or diffuse within the visualized surface area. Abnormal lymph nodes, calcifications, and visibility of posterior border were marked as present or absent. Using a system 0–48 based on similar characteristics, A. Hočevar and colleagues determined that a cut-off score of 17 resulted in a specificity of 98.7% and a sensitivity of 58.8% [37]. This supports that SGUS offers similar diagnostic accuracy when compared to more invasive conventional testing methodologies.

Sonoelastography is a newer technique being studied as an additional semi-quantitative criterion for diagnosis of SS. Unlike traditional SGUS, elastography measures displacement and elasticity of the target tissue by using color spectrum and strain rates. As SS leads to progressive inflammation and fibrosis, there can be loss of parenchymal elasticity. In a study comparing 58 patients with SS to 24 healthy volunteers, there was a statistically significant difference found between SS and control groups for elastographic scores and strain ratios [38]. Sonoelastography is also being studied and utilized in the assessment of breast lesions, thyroid nodules, and liver fibrosis [39]. It will be seen whether continued advancement in this field will allow for more accurate diagnosis of SS without the need for invasive procedures.

## 7. Giant Cell Arteritis

US is a mainstay imaging modality used for vascular pathology such as thromboembolism and peripheral arterial disease. In recent years, it has gained acceptance for the diagnosis of giant cell arteritis (GCA), a large vessel vasculitis affecting the aorta, its major branches, and cranial vessels. It rarely affects people under the age of 50. Patients often present with headache, scalp tenderness, jaw and tongue claudication, dysphagia, cough, and diplopia. However, its most feared complication is blindness [40]. Early identification and intervention with high dose glucocorticoids can prevent permanent vision loss [41].

Diagnosis is generally made by clinical history, physical examination, elevated inflammatory markers such as erythrocyte sedimentation rate, and c-reactive protein. The gold standard diagnostic test is a biopsy of the temporal artery to evaluate for pan-arteritis and presence of mononuclear cell infiltration, multinucleated giant cells and intimal hyperplasia with concentric occlusion [40]. Unilateral sampling is usually sufficient, but contralateral specimen may be needed in the setting of high clinical suspicion. Segmental involvement and inadequate length of vessel contribute to the false negative rate, as the mean pooled sensitivity of unilateral temporal artery biopsy is 86.9%. Specificity is assumed to be 100%, as clinicians would generally not withhold treatment in such a scenario [42]. The decision to pursue bilateral biopsy is often complex, as one must weigh the impact on therapy with the invasive nature of the procedure.

The color Doppler US possesses high spatial resolution of 0.1 mm, therefore, it can offer non-invasive visualization of the temporal artery and other extra-cranial vessels [43]. The finding highly correlated with GCA is a circumferential hypoechoic area surrounding the vessel lumen. This “halo sign” likely reflects edema of the vascular wall, and measures 0.3 to 1.2 mm in diameter [44]. When compared to temporal artery biopsy, the presence of a unilateral halo sign had a sensitivity of 68% and a specificity of 91%. Bilateral halo signs achieved a higher specificity of 100% but a lower sensitivity at 43% [45]. As US technologies progress and technician training becomes standardized, use of a color Doppler may become more widespread in the diagnosis of GCA.

## 8. Polymyalgia Rheumatica

Polymyalgia rheumatica (PMR) is a common inflammatory disease of the elderly. It classically presents with proximal muscle pain and stiffness primarily of the shoulder and pelvic girdles. Diagnosis is often challenging, as there are no reliable diagnostic tests, and presenting symptoms can also mimic a number of other rheumatologic conditions. While elevated inflammatory markers are considered a hallmark of this condition, they are non-specific and often elevated at baseline in the susceptible population [46]. Clinicians frequently rely on corticosteroid response as a diagnostic clue, though this approach is not without its pitfalls. Corticosteroids exhibit potent anti-inflammatory effects across various inflammatory arthritides, so clinical improvement may not confer a definitive diagnosis of PMR. Long term use of glucocorticoids can be associated with significant morbidity, thus highlighting the importance of a definitive diagnosis.

The need for more specific biomarkers has led to the introduction of MSUS at the point of care. In 2012, The American College of Rheumatology (ACR) developed provisional criteria for the diagnosis of PMR with and without the use of US. The study found that adding MSUS improved the specificity from 78% to 81%, and it offered greater discriminant ability for mechanical shoulder pathologies, such as bilateral rotator cuff tendinopathy, adhesive capsulitis, and glenohumeral osteoarthritis [47].

**Figure 8:**
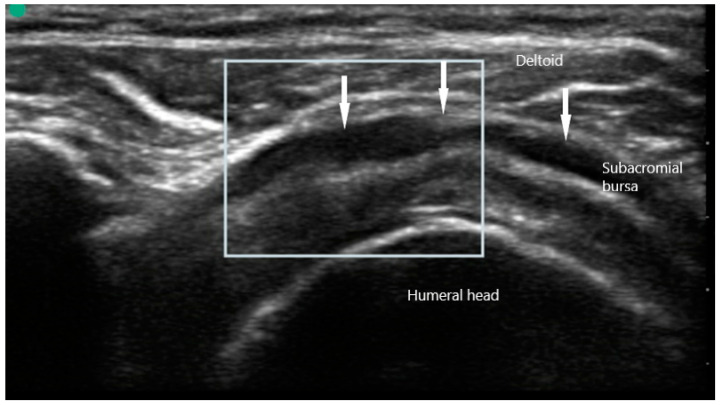
Subacromial bursitis (arrows) overlying supraspinatus in polymyalgia rheumatica.

US findings in patient with PMR frequently reveal inflammatory changes of the affected joints, along with peri-articular structures. Shoulder pain may manifest on MSUS as subacromial bursitis (Figure 8) and is seen as a hypoechoic collection superficial to the supraspinatus. Other findings may include bicipital tenosynovitis and glenohumeral effusion. Pain of the hip girdle commonly reveals hip effusion and trochanteric bursitis. These findings are non-specific, but may offer crucial evidence when taking into the context the wider clinical picture. While all these US findings may be present due to mechanical etiologies, bilateral involvement as well as severity of the inflammation can suggest an inflammatory component.

## 9. Crystalline Arthropathies

Crystalline arthropathies are among the most common forms of inflammatory arthritides [48]. They are a group of disorders in which crystals, such as urate and calcium pyrophosphate, can accumulate in the articular and periarticular tissues leading to inflammation, joint pain, and damage. Other crystals such as calcium hydroxyapatite and calcium oxalate can rarely be implicated. These crystalline pathologies are often indistinguishable in clinical presentation, especially when large joints are involved. Arthrocentesis with synovial fluid analysis using polarized microscopy has long been the gold standard [49]. Dual-energy computed tomography (DECT) is a relatively recent development in imaging and diagnosis of gouty arthritis. The principle behind its use is to differentiate materials based on their relative absorption of x-rays at different photon levels. Post processing of these images typically depicts three-dimensional rendering with monosodium urate crystals appearing green. A meta-analysis of the diagnostic accuracy of DECT by Ogldie et al. showed a sensitivity of 87% and a specificity of 84% [50]. Some disadvantages of DECT are its relatively high doses of radiation, as well as its inability to identify other crystals, such as calcium pyrophosphate. The advantage of using MSUS is the ability to detect the distribution of crystals within a joint, and ultimately help distinguish between these different clinical entities.

### 9.1. Gout

Gout is the most prevalent form of inflammatory arthritis, and is characterized by tissue deposition of monosodium urate (MSU) crystals [51]. It is more common in men than women, and has increasing prevalence with age. Other risk factors include obesity, high caloric diet, and comorbidities such as chronic kidney disease. In the United States, approximately 3–4% of the adult population is affected [52]. Serum urate levels are usually elevated, though definitive diagnosis can only be made by detection of rod-shaped negatively birefringent crystals on polarized microscopy. It can present as acute episodes of severe inflammatory monoarthritis or oligoarthritis. The most well-known clinical presentation is podagra, which is an attack involving the 1st metatarsal phalangeal joint. Chronic arthritis with devastating joint destruction may occur, and can be mistaken for other forms of inflammatory arthritis. Tophi are deposits of MSU crystals in periarticular tissue, and may be present in untreated later stages of the disease.

**Figure 9:**
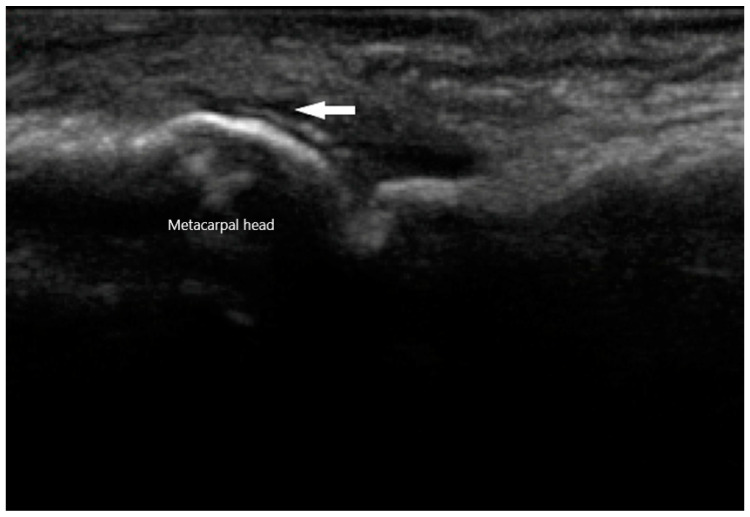
Double contour sign (arrow) of metacarpal head in gout.

MSU crystals can be seen easily via US as they reflect US waves. They typically appear to be hyperechoic on the superficial margin of the cartilage in either a homogenous or irregular distribution, typically referred to as a “double contour” sign [53] (Figure 9). Any joint can exhibit this finding, but it is most commonly seen in the suprapatellar short axis view of the knee when placed in a flexed position. This ultrasonographic finding is highly suggestive of gout, with a specificity of 97.3% and a positive predictive value of 93.3%. The sensitivity and negative predictive value, on the other hand, is low at 36.8% and 60% respectively [54]. When using the “double contour” sign, one common pitfall is the occasional smooth, hyperechoic appearance of the surface of hyaline cartilage. While it may appear as a “double contour”, only the part of the cartilage that is perpendicular to the US beam will be visible. In contrast, MSU deposition exhibits a more diffuse and irregular pattern. Gouty bone erosions are more effectively detected via US compared to plain radiographs, with respective rates of 67% and 28% for the first metatarsal phalangeal joint [55].

Synovitis in gout may be similar to other forms of inflammatory arthritis such as RA and SpA. While synovitis in RA tends to be hypoechoic and homogeneous, synovitis in gout can occasionally appear heterogeneous or hyperechoic. Tophi are another important US finding in the diagnosis of gout. They can be found within or surrounding any joint, but are seen most commonly in the olecranon bursa, knees, hands, and ears. Its presence suggests advanced disease with a high urate burden. Tophi appear as a hyperechoic amorphous substance with an anechoic rim. They may also exhibit acoustic shadowing due to its high density and impedance [56].

### 9.2. Calcium Pyrophosphate Dihydrate Deposition (CPPD) Disease

CPPD disease is another common crystalline arthropathy, in which calcium pyrophosphate crystals are deposited into various tissues. There are several different clinical presentations, including acute inflammatory arthritis, chronic inflammatory arthritis, and degenerative arthritis. Acute arthritis is also called “pseudogout”. As suggested by its namesake, pseudogout is frequently mistaken for gout given its mono-arthritic or oligo-arthritic distribution, episodic occurrence, and severity. Definitive diagnosis can only be made by identification of rhomboid shaped positively birefringent crystals on polarized microscopy. Chronic inflammatory arthritis may affect small joints such as the hands and feet, presenting as “pseudo-rheumatoid arthritis” [57]. Degenerative arthritis can manifest in joints such as the wrists or ankles, which are ordinarily spared by osteoarthritis. Most patients with this disease are elderly, as a cross-sectional study involving 2157 veterans showed a point prevalence of 5.2 per 1000, and an average age of 68 years [58].

**Figure 10:**
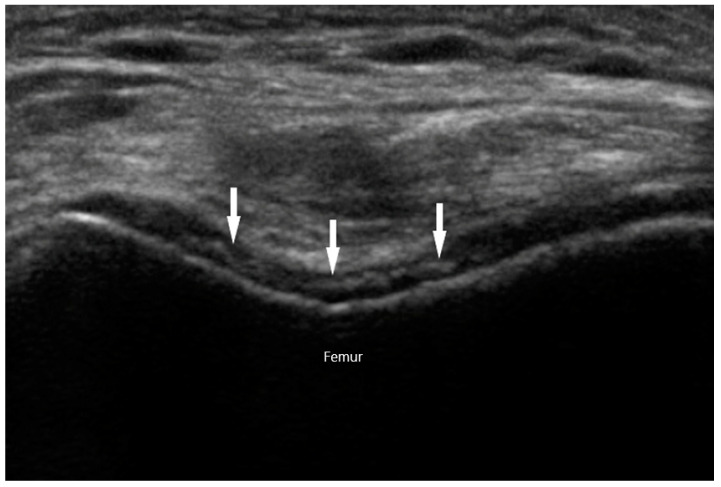
Chondrocalcinosis (arrows) within knee cartilage in calcium pyrophosphate deposition disease.

Chondrocalcinosis refers to articular or periarticular deposition of calcium crystals. It can be detected by various imaging modalities, including radiographs, CT, MRI, and US. Chondrocalcinosis on radiographs is most commonly seen in the wrists, knees, or pelvic symphysis. They appear as amorphous radiopaque material within the joint or surrounding fibrous tissues. Sonographic findings suggestive of CPPD arthropathy include isolated hyperechoic amorphous areas, hyperechoic bands within the cartilage, calcifications within the tendon, or hyperechoic nodular deposits within bursas. In contrast to the gouty “double contour” sign on cartilaginous surfaces, the hyperechoic calcifications in CPPD are seen within the hyaline cartilage itself (Figure 10). In a study examining knees in 74 patients with CPPD, Grassi et al. discovered that calcifications in hyaline cartilage were detected by US at a rate of 59.5%, compared to 45.9% by conventional radiography. Specificity for the finding was 100% [59].

## 10. Limitations and Uncertainties of MSUS

There are several disadvantages to MSUS when compared to other imaging modalities in the diagnosis and management of rheumatic diseases. Conventional radiographs, computed-tomography, and MRI rely on trained technicians to obtain the desired images for radiologists’ interpretation. In contrast, MSUS is usually performed at the point-of-care by the treating rheumatologist. Therefore, the diagnostic yield of US is highly operator dependent. While there is no universal certification, there have been initiatives to improve standardization of education with programs such as Ultrasound School of North American Rheumatologists (USSONAR) and the Musculoskeletal Ultrasound Certification in Rheumatology (RhMSUS).

Another limitation of MSUS is the limited visual field. While radiographs and MRI offer evaluation of the entire anatomical area, US can only be applied to one joint at a time. For example, one MRI scan of the hand can show every joint with the associated soft tissues. In order to collect the same information with US, clinicians have to assess every single joint separately in longitudinal, transverse, and occasional lateral views. Such an endeavor is extremely time-intensive and inefficient.

Body habitus can also present a daunting obstacle for accurate US assessment. Image resolution and quality can be compromised in obese patients, due to the limited visual depth. This problem is compounded when evaluating deep structures such as the hip or SI joint. A linear probe, which can provide approximately 8 cm of depth may be insufficient, but the deeper penetration of a curvilinear probe sacrifices image resolution.

## 11. Conclusions

Since the inception of A-mode and 2D mode, there has been substantial progress in US technology in recent decades. Improvements in imaging quality and adoption of color and power Doppler has significantly improved diagnostic accuracy at the point of care. Its other advantages include accessibility and safety, given the lack of ionizing radiation and absolute contraindications. This has contributed to its widespread use for medical applications worldwide, as the market for US equipment is projected to reach $8.3 billion by 2023. Numerous medical specialties routinely employ US for diagnostic and therapeutic applications. Sonoelastography and three-dimensional US are newer breakthroughs that are currently being investigated for clinical use [60]. Its adoption in rheumatology practice has been a crucial advancement in the field, especially given the scarcity of definitive diagnostic markers for many rheumatic diseases. With continued innovations in US technology, rheumatology may continue to be a prime beneficiary.
